# Semi-automated ultrasound guidance applied to nasogastrojejunal tube replacement for enteral nutrition in critically ill adults

**DOI:** 10.1186/s12938-018-0452-1

**Published:** 2018-02-07

**Authors:** Ying Li, Yu Ye, Yang Mei, Haiying Ruan, Yuan Yu

**Affiliations:** 1 Department of Critical Care Medicine, Second People’s Hospital of Shenzhen, Shenzhen, 518035 People’s Republic of China; 2 Department of Neurosurgery, Longgang Central Hospital of Shenzhen, Shenzhen, 518116 People’s Republic of China

**Keywords:** Enteral nutrition, Nasogastrojejunal tube, Semi-automated ultrasound, Feeding tube placement

## Abstract

**Background and objective:**

At present, the enteral nutrition approaches via nose and duodenum (or nose and jejunum) are the preferred method of nutritional support in the medical engineering field, given the superiority of in line with physiological processes and no serious complication. In this study, the authors adopted saline as the acoustic window, and gave enteral nutrition support to critically ill patients, via the nasogastrojejunal approach guided by semi-automated ultrasound. These above patients benefited a lot from this kind of nutrition support treatment, and we aimed to report the detailed information.

**Methods:**

Critically ill patients (n = 41) who had been treated with hospitalized intestine nutrition were identified. The Apogee 1200 ultrasonic diagnostic apparatus, and nasogastrojejunal tubes were adopted to carry out intestine nutrition treatment guided by semi-automated ultrasound. In order to confirm the specific positions of cardia, gastric body, antrum of stomach, and pylorus, the semi-automated ultrasound was utilized to probe the stomach cavity. And then, the ultrasonic probe was placed in the cardia location, and the nasogastrojejunal tube was slowly inserted through the metal thread. After operation, the nursing service satisfaction of patients and mean operation time were calculated, respectively.

**Results:**

All the patients were treated with enteral nutrition via nasogastrojejunal tube, and the whole procedure was under the guidance of semi-automated ultrasonography. The end of the feeding tube is attached to the surface of the stomach with a greater curvature, which can be bent on account of a no gastric peristalsis squeeze function, and thereby were prevented from entering into the antrum and pylorus locations. After this procedure, the mental thread was taken out, and the tube was pushed forward by a “drift” approach in order to allow it to enter into the intestine. The total nursing service satisfaction of patients was 90.24%, and the total incidence of adverse reactions was 17.07%.

**Conclusions:**

In summary, the application of saline can be taken as sound window, and the metal wire as the tracking target, the bedside nasogastrojejunal tube guided by semi-automated ultrasound is an effective feeding tube placement method, with relatively good clinical application value in medical engineering.

## Background

Nutrition support is one major development of clinical medicine in the twentieth century, and has become an indispensable constituent part in the treatment of critically ill patients, in order to alleviate the nutritional deficit [1, 2]. Enteral nutrition has achieved significant advances in decades, and is beneficial for the patients who have functional guts but can not meet their nutritional requirements via normal diet, on account of cancer, HIV, stroke, multiple sclerosis, dementia, etc. [3–5]. This kind of enteral feeding can be delivered by means of various approaches, including nasogastric tube, percutaneous endoscopic gastrostomy, jejunostomy. In general, during the period of enteral nutrition, the providers also need to assess the nutritional status, and evaluate the nutritional requirements of patients [6]. Besides, the development of enteral nutrition also requires multidisciplinary teams, such as the extended roles for dietitians and nurses, etc. [7]. In addition, more and more serious aging society, various diseases mentioned above, the swallowing difficulties and malnutrition resulted from various complications, are all the main reasons why rapidly increasing enteral nutrition is needed [8, 9].

At present, the enteral nutrition approaches via nose and duodenum (or nose and jejunum) are the preferred method of nutritional support in medical engineering, given the superiority of in line with physiological processes and no serious complication [10, 11]. In this study, the authors adopted saline as the acoustic window, and gave enteral nutrition support treatment to critically ill patients, via the nasogastrojejunal approach guided by semi-automated ultrasound. These above patients benefited a lot from this kind of nutrition support, and we reported the detailed information as followed.

## Methods

### Clinical data of the identified patients

**Fig. 1:**
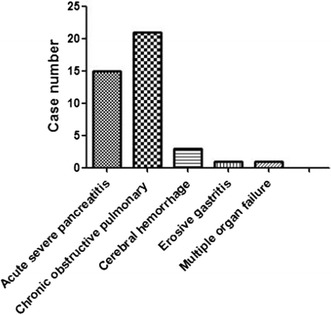
Distribution of primary diseases involved in this research

**Fig. 2:**
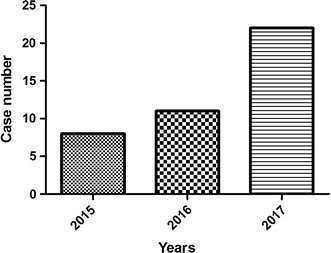
Case number per year from 2015 to 2017

This research was approved beforehand by the institution ethics committee in our hospital. According to the relevant regulations of ethics, the informed consent of patients had been obtained before investigation. 41 critically ill patients who would be treated with hospitalized intestine nutrition were identified in our department, from February 2015 to January 2017. In detail, 34 males and 7 females, the age ranged from 21 to 86 years old. 15 cases suffered from primary acute severe pancreatitis, 21 cases suffered from chronic obstructive pulmonary disease and respiratory failure, 3 cases suffered from cerebral hemorrhage, 1 case suffered from erosive gastritis, 1 case suffered from multiple organ failure, as shown in Table 1 and Fig. 1. And, Fig. 2 illustrated the case number per year from 2015 to 2017.

**Table 1:** General information of patients identified in this study

Diseases	Number	Gender (male/female)	Average age
Primary acute severe pancreatitis	15	13/2	48.20 ± 7.80
Chronic obstructive pulmonary disease	21	18/3	59.22 ± 7.77
Cerebral hemorrhage	3	1/2	68.21 ± 6.11
Erosive gastritis	1	1/0	35
Multiple organ failure	1	1/0	62

### Instrument and methods

The Apogee 1200 portable ultrasonic diagnostic apparatus (Japan), and Ch 12 nasogastrojejunal tubes (Manufacturer: Bai Tong in China) were adopted to carry out intestine nutrition treatment guided by semi-automated ultrasound. These included patients fasted for 6–8 h, and then received gastrointestinal decompression treatment for 1 h in order to vent the gas and food residues in the stomach, so that the images could be clearly displayed. The patients kept sober and took semi-recumbent, in order to make them coordinate work during treatment. The routine abdomen ultrasound was utilized to probe the stomach cavity, in order to confirm the specific positions of cardia, gastric body, antrum of stomach, and pylorus. And then, the ultrasonic probe was placed in the cardia location, and the nasogastrojejunal tube was slowly inserted through the metal thread. The ultrasonic diagnostic apparatus could capture the image of metal thread, and the intubationist slowly injected pre-heated physiological saline 300–400 ml. Afterwards, the ultrasonic probe was used to perform fan-shaped scan, with the purpose of knowing the specific location of nasogastrojejunal tube and the status of gastric motility. The intubationist slowly rotated the guide wire to make it follow the gastric motility, and at the same time slowly pushed the tube. The nasogastrojejunal tube straightly went into the pylorus, and was forwarded smoothly. When the intubationist felt empty, the tube entered into duodenum. The location of ultrasonic probe was adjusted, the duodenal bulb could be observed, and the ultrasonic echo of metal thread was stronger. And then the thread was pushed forward to another 110–120 cm. The bedside abdomen radiographs were obtained to confirm whether the tube position was proper, and then the guide wire was took out. And then, the operator made sure that the patient was in a good condition, and then another 20 ml saline was injected again to assure the tube was unobstructed, the gas over the water tone could be heard by umbilical auscultation. At last, the enteral nutrition pump was connected with the tube. After operation, the nursing service satisfaction of patients and mean operation time were calculated, respectively.

## Results

### General information

**Fig. 3:**
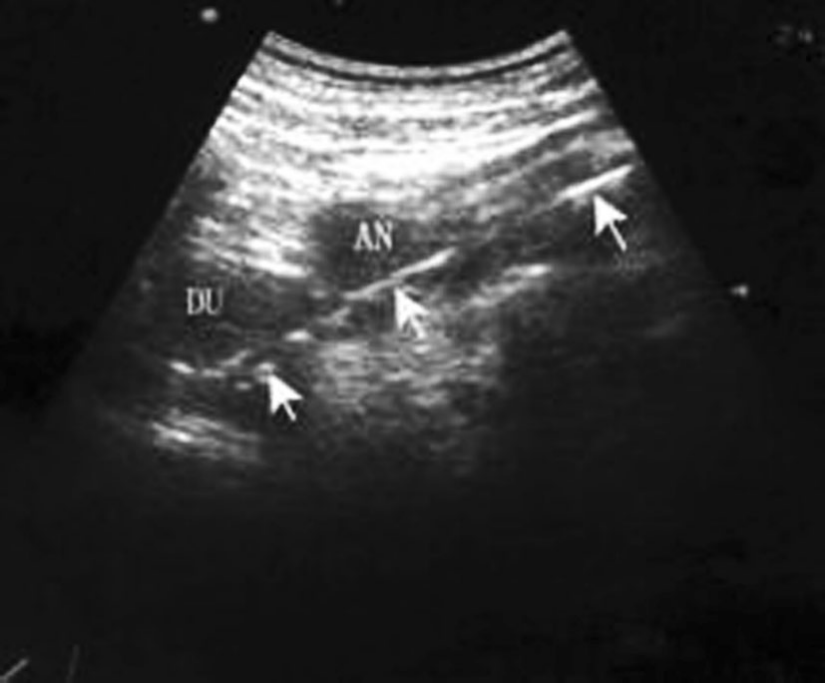
Captured image when the nasogastrojejunal tube entered into pylorus and duodenal bulb

All the patients were treated by using nasogastrojejunal tubes via guidance of semi-automated ultrasonography, and the status of the patients during operations were all good. 40 cases were successfully implanted, 1 case failed. The failed case suffered from multiple organ failure, and the gastric motility disappeared. The end of the feeding tube is attached to the surface of the stomach with a greater curvature, which can be bent on account of a no gastric peristalsis squeeze function, and thereby prevented from entering into the antrum and pylorus locations. After this procedure, the mental thread was taken out, and the tube was pushed forward by a “drift” approach in order to allow it to enter into the intestine. Nevertheless, the operation time lasted longer, and the nasogastrojejunal tube was placed into the intestine after 2 h. And, Fig. 3 illustrated the captured image when the nasogastrojejunal tube entered into pylorus and duodenal bulb.

### Average operation time of intubation

The average operation time of intubation in this study was displayed in Table 2. 15 cases suffered from primary acute severe pancreatitis, and the average operation time was 48.20 ± 12.50 min. 21 cases suffered from chronic obstructive pulmonary disease, and the average operation time was 26.80 ± 6.52 min. 3 cases suffered from Cerebral hemorrhage, and the average operation time was 44.21 ± 4.59 min. 1 patients with Erosive gastritis, and 1 case failed.

**Table 2:** Average operation time of intubation in this study

Diseases	Number	Average operation time (min)
Primary acute severe pancreatitis	15	48.20 ± 12.50
Chronic obstructive pulmonary disease	21	26.80 ± 6.52
Cerebral hemorrhage	3	44.21 ± 4.59
Erosive gastritis	1	32
Multiple organ failure	1	Failed

### Patients’ satisfaction degree

**Fig. 4:**
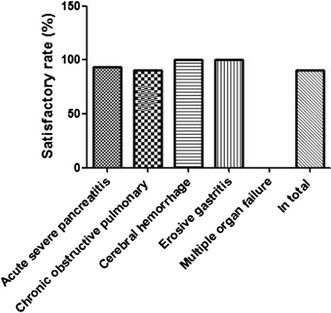
Nursing service satisfaction of patients suffering from different diseases

Besides, we made another questionnaire survey to investigate the patients’ satisfaction degree of nasogastrojejunal tube, and the result was illustrated in Table 3 and Fig. 4. The total nursing service satisfaction of patients was 90.24%. The satisfactory degree of patients with primary acute severe pancreatitis was 93.33%, the satisfactory degree of patients with chronic obstructive pulmonary disease was 90.48%, the satisfactory degree of patients with cerebral hemorrhage and erosive gastritis both were 100%. In general, the total nursing service satisfaction appeared high, more than 90%, which suggested that this kind of technique could be acceptable very well. Nevertheless, several serious complications occurred, on account of the diseases or the operators, and the nursing service satisfaction of these patients decreased.

**Table 3:** Nursing service satisfaction of patients identified in this study

Diseases	Number	1^a^	2^b^	3^c^	Total satisfactory (%)
Primary acute severe pancreatitis	15	6	8	1	93.33
Chronic obstructive pulmonary disease	21	7	12	2	90.48
Cerebral hemorrhage	3	1	2	0	100
Erosive gastritis	1	0	1	0	100
Multiple organ failure	1	0	0	1	0
In total	41	14	23	4	90.24

^a^Great satisfaction; ^b^ satisfaction; ^c^ dissatisfaction

### Incidence of adverse drug reactions

**Fig. 5:**
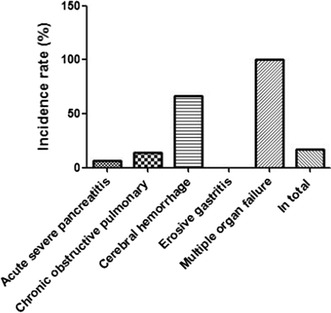
Adverse reactions resulting from different diseases

In addition, in terms of incidence of adverse drug reactions among the identified patients in this study, we calculated them and listed in Table 4 and Fig. 5. The main adverse drug reactions in this research were stomachache, headache, nausea and vomiting. The incidence of adverse reactions of primary acute severe pancreatitis was 6.67%, the incidence of chronic obstructive pulmonary disease was 14.29%, the incidence of cerebral hemorrhage was 66.67%, the incidence of erosive gastritis was 0%. At last, the total incidence of adverse reactions was 17.07%.

**Table 4:** Incidence of adverse reactions among the identified patients in this study

Diseases	Number	1^a^	2^b^	3^c^	Total rate (%)
Primary acute severe pancreatitis	15	0	0	1	6.67
Chronic obstructive pulmonary disease	21	0	0	3	14.29
Cerebral hemorrhage	3	0	1	1	66.67
Erosive gastritis	1	0	0	0	0
Multiple organ failure	1	0	0	1	100
In total	41	0	1	6	17.07

^**a**^Stomachache; ^**b**^ headache; ^**c**^ nausea and vomiting

## Discussion

With the rapid development of medical engineering, the medical care of patients with critical illness has become increasingly complicated [12–14]. And the nutrition support has long been recognized as supportive therapy in critical care. In recent decades, the clinical nutrition has been evolving all the time, and it has been deemed as a form of therapy, rather than just a way of delivering nutrition [15–17]. Because several nutrition might exert therapeutic effects via immunomodulation or liver protection, etc. For instance, among cancer patients, the enteral nutrition contributes to decrease the postoperative complications and prolong survival time [18]. At present, the application of immune-enhancing enteral nutrition is an integral part of surgical guidelines. On the basis of latest ESICM clinical practice guidelines, the initiating enteral nutrition has been recommended to be provided within the first 24–48 h after intensive care unit admission, if the patients can not eat by themselves [19, 20].

The main approaches of enteral nutrition are various, but the commonly used and noninvasive method is nasogastrojejunal tube. The traditional method of intubation tube always depends on X ray or gastroscope, or only relies on the operator experience. When the intubationist just operates by his own experience, the success rate of intubation appears lower [21, 22]. When the intubationist operates under the guidance of X ray, the patients have to be moved, but the majority of patients are critically ill in the intensive care unit and are hard to be carried, especially for the patients who need mechanical ventilation [23, 24]. Nasogastrojejunal tube insertion is based on minimally invasive catheterization procedure that is combined with ultrasound guidance. This semi-automated device pertains to a class of medical imaging assisted equipment that can help patients in terms of enteral nutrition [25].

On the other hand, the superiorities of intubation guided by semi-automated ultrasound are as follows. (1) The operation by the bed is permitted, without moving the patient. And the operator can observe the location of nasogastrojejunal tube. (2) The operation guided by semi-automated ultrasound do not bring about radioactive injury, and the painful feeling of patients was smaller when receiving this noninvasive method. (3) It is cost-effective method, when compared with digital subtraction angiography and gastroscope. In addition, there are several points need to be addressed during implementation. (1) Before operation, the abrosia for 6–8 h is needed, as well as gastrointestinal decompression for 1 h, in order to exclude interference resulted from food residue and gas in the stomach, and thereby to improve the image quality, to clearly display the tube position, and to reduce the failure rate of operation. (2) When the tube enters into gastral cavity, another 300–400 ml saline should be injected into tube. The saline should be placed in the incubator and be heated to 37 °C, to avoid unnecessary stimulation. The injected saline forms an acoustic window, so that the nasogastrojejunal can be clearly displayed. (3) The tube should be carried forward, with the help of gastric peristalsis, and this is also the pivotal point. The stomach cavity has two physiological curves, namely, stomach angle and pyloric region. The metal wire always remains extended position, and it is difficult to go through the two bending angles. When the peristaltic wave gets through the two physiological bending, the bending angles disappear, and the extrusion force pushes forward the tube, until entering into duodenum. The only one failure case attributed to the disappearance of gastric motility in the patient suffering from multiple organ failure.

## Conclusion

To sum up, the application of saline can be taken as sound window, and the metal wire as the tracking target, the bedside nasogastrojejunal tube guided by semi-automated ultrasound is an effective feeding tube placement method, with relatively good clinical application value in medical engineering.
